# The Corvis ST analysis of underaged versus adults’ healthy eyes with comparable tomography detects softer corneas in children and adolescents as opposed to adults

**DOI:** 10.1038/s41598-026-52447-1

**Published:** 2026-05-18

**Authors:** Elias Flockerzi, Tim Berger, Yaser Abu Dail, Jessica Obst, Albéric Sneyers, Maximilian Berger, Max Bofferding, Julian Kahlert, Tommaso Paoletti, Paul Teping, Paul Kohlhas, Berthold Seitz

**Affiliations:** Department of Ophthalmology, Saarland University Medical Center, Kirrberger Straße, Building 22, 66421 Homburg, Germany

**Keywords:** Corneal biomechanics, Corvis, Tomography, Dynamic corneal response, Diseases, Health care, Medical research

## Abstract

This cross-sectional cohort study aimed to analyze whether corneal biomechanics differ between healthy eyes of different age, even if they appear tomographically identical. All volunteers underwent corneal Scheimpflug imaging (Pentacam) and biomechanical examination (Corvis ST, CST both Oculus, Germany). Three groups (G) were formed according to age: (G1) children aged 3–10 years (*n* = 250, 7.7 ± 1.9 years (mean ± standard deviation)), (G2) adolescents aged 11–20 years (*n* = 350, 15.3 ± 2.9 years) and (G3) adults (*n* = 100, 48.7 ± 13.8 years). Main tomographic outcome parameters were: flat anterior (K1F)/posterior (K1B) and steep anterior (K2F)/posterior (K2B) meridians, thinnest corneal thickness (TCT) and maximal anterior keratometry (Kmax). Considering biomechanics, these were deformation amplitude ratio 2 mm (DA ratio 2 mm), integrated radius (IR), Ambrósio Relational Thickness horizontal (ARTh), stiffness-parameter (SP-A1) and corneal velocity at inward applanation (A1 velocity), stress-strain-index (SSI), Corvis Biomechanical Index (CBI), Biomechanical E-Staging (BEST) and the CST-derived non-contact (IOPnct) and biomechanically adjusted intraocular pressure (bIOP). The groups were compared with Kruskal–Wallis one-way ANOVA and Dunn’s post test. The three groups G1–G3 were tomographically comparable (*p* > 0.05 for K1F/K1B/K2F/K2B/TCT/Kmax). Biomechanically, there was no difference for DA ratio 2 mm and ARTh between G1–G3. Significant differences were found for SP-A1 (G1 < G2< G3, *p* = 0.0125), IR (G2 < G3, *p* = 0.0288), A1 velocity (G1 < G2< G3, *p* = 0.0027), SSI (G2 < G1< G3, *p* < 0.0001), CBI (G3 < G2< G1, *p* = 0.001), BEST (G3 < G2< G1, *p* = 0.0015), IOPnct (G3 < G1< G2, *p* = 0.0006) and bIOP (G3 < G2< G1, *p* < 0.0001). There exist small, yet significant biomechanical differences in CST measurements between tomographically comparable healthy corneas in dependence of age indicating biomechanically weaker corneas in children than in adults.

## Introduction

The tomographical examination of the human cornea includes measurements of anterior and posterior corneal curvature and corneal thickness^1^. The biomechanical properties of the human cornea can be examined by applying a standardized air-puff on it and interpreted on the basis of the following corneal deformation behavior. In contrast to healthy corneas, there is a greater deformation response and less corneal resistance to deformation in keratoconus (KC), which is the most common keratectasia^2^. Thus, the resulting deformation may help in differentiating between healthy and KC corneas^3^. The measurement itself may be provided by the Corneal Visualization Scheimpflug Technology Corvis ST (CST, Oculus, Wetzlar, Germany), which measures both single and combined biomechanical parameters and different intraocular pressure (IOP) values during the deformation response of the cornea, which is characterized by an inward movement, passing an inward applanation (A1), a concave phase and followed by an outward movement including an outward applanation (A2) prior to returning in its initially convex shape. The Corvis Biomechanical Index (CBI) is a logistic regression algorithm that combines different individual biomechanical parameters to differentiate between healthy and KC corneas^3,4^. It is reported to be composed of (1) deformation amplitude ratio 2 mm (DA ratio 2 mm, the ratio between central deformation and deformation 2 mm from the center of the cornea), (2) integrated inverse radius (IR, the sum of the inverse radii of the concave state between the first (A1) and second (A2) applanation), (3) Ambrósio relational thickness horizontal (ARTh, the ratio of corneal thickness at thinnest corneal thickness to the pachymetric progression index), (4) stiffness parameter A1 (SP-A1, the ratio of the force of the air applied to the corneal displacement) and (5) A1 velocity (the velocity of the corneal apex at inward applanation A1). The biomechanical E-staging (BEST) is based on the linearized term of the CBI^5^ providing a biomechanical keratectasia severity staging and progression assessment.

The CST measurement results depend on the viscoelastic properties of the human cornea. Factors that may further influence corneal biomechanical properties include IOP, ametropia, gender and age. One study reported corneal stiffness to be reduced in myopia and increased in hyperopia compared with emmetropia in Chinese preschool children from four to six years^6^. Another study based on 733 corneas of Indian children from six to 18 years reported that the high myopia group (-6 to -9 diopters manifest refraction spherical equivalent MRSE) showed a lower biomechanical stiffness than the moderate (-3 to -6 diopters MRSE) or the low myopia group (0 to -3 diopters MRSE^7)^. This observation of a decreased biomechanical resistance to corneal deformation in myopia was corrobated in 1,645 Chinese university students^8^. A recent meta-analysis based on CST-based biomechanical studies concluded that corneas of highly myopes (more than − 6 diopters) deformed slower during first applanation, faster during second applanation and showed a greater deformation amplitude than emmetropes^9^. However, this meta-analysis included only one study with children aged nine to eleven years^9^. Consequently, a progressive biomechanical weakening of the cornea has to be expected with increasing myopia^7^.

Age is another determinant of corneal biomechanics, as ageing leads to a stiffening of the human cornea^8,10^ by (1) an increase in the number of collagen molecules^11^, (2) a three-dimensional growth of collagen fibers in the corneal stroma^12^, (3) glycosylation leading to an expansion of the spacing between molecules and (4) changing proteoglycan composition of the inter-fibrillary matrix^11^.

Gender is another factor that may affect corneal biomechanics and it has been reported that estrogen can reduce corneal stiffness^9^. Nevertheless, the reported data is heterogeneous: Data based on Ocular Response Analyzer measurements (Reichert Instruments, Depew, New York, United States of America) reported a significantly lower corneal hysteresis (CH) and corneal resistance factor (CRF) in 175 healthy men (mean age: 28.6 ± 6.6 years, mean refractive error − 3.6 ± 2.4 Diopters) compared to 175 healthy women (mean age: 27.1 ± 5.9 years, mean refractive error − 3.6 ± 2.5 Diopters^13)^ contrasting with a study reporting a significantly lower CH and CRF in women than men in an older study population consisting of 400 healthy subjects (mean age: 58.8 ± 17.2 years^14)^. For CST measurements, one study found a significantly higher A1 velocity in females than males among 652 keratorefractive surgery candidates (mean age: 28 ± 5 years), thus indicating a biomechanically weaker cornea in females^15^. This contrasts with a study that investigated corneal biomechanics in 1645 healthy university students (mean age: 19.08 ± 0.9 years, mean spherical equivalent − 3.7 ± 2.4 Diopters), who concluded that the highest concavity deflection amplitude, highest concavity peak distance, maximal deformation amplitude ratio and A1 velocity were significantly higher in men than in women^8^. Finally, another study did not find any significant difference for any biomechanical CST parameter among 158 healthy adults between males and females^16^.

Considering CST-measurements, the CST-derived CBI itself has been developed based on a first database including 227 healthy (patients’ mean age 37 ± 12 years) and 102 KC corneas (patients’ mean age 43 ± 17 years) and validated based on a second database including 251 healthy (patients’ mean age 32 ± 12 years) and 78 KC corneas (patients’ mean age 37 ± 17 years^4)^. The reference population in the CST software for both healthy and KC corneas (*n* = 5500) contains less than 10% children and adolescents (personal communication with Dr. Sven Reisdorf, Oculus). Consequently, one first question is whether the CBI, that has been validated primarily for people in their mid-thirties to mid-forties, can also be used for younger patients with healthy eyes. Secondly, there is a lack of reference data, particularly of children and adolescents. Because biomechanically softer corneas have been reported in healthy children^6,7,11,17^, the current study aimed to analyze, whether CST parameters may detect differences in corneal biomechanical properties according to age in tomographically comparable healthy corneas. Consequently, the current study investigated corneal biomechanical properties as provided by the CST in healthy corneas of Caucasian children, adolescents and adults with comparable tomography and manifest refraction spherical equivalent.

## Methods

Healthy volunteers were examined first with corneal Scheimpflug imaging (Pentacam high resolution) and thereafter with the CST (both Oculus, Wetzlar, Germany) in automatic release mode by an experienced technician. The local ethics committee was informed and approved this cross-sectional cohort study (Ethikkommission bei der Ärztekammer des Saarlandes, approval number 149/21) that was conducted in accordance with the Declaration of Helsinki. All volunteers or their respective legal guardians gave written consent for the analysis of their data. Ophthalmologic patients with prior surgery, a chronic ocular condition, regular application of topical therapy in form of eye drops or contact lens wearers were excluded. After taking the medical and ophthalmological history, slit lamp examination of the volunteers’ eyes and visual acuity testing, one eye of each healthy volunteer without diagnosis of systemic conditions was randomly selected for evaluation. Initially, there were 1000 healthy volunteers from 3 to 21 years of age that participated in this study. As the feasibility and repeatability of non-contact-tonometry and CST measurements may be affected by challenges in fixation and potential blepharospasm especially in pediatric volunteers, only measurements with an “OK“ quality score were accepted, which led to the exclusion of 400 participants from 3 to 21 years of age despite repeated measurements (maximum: three measurements per eye). The measurements were conducted between 09.00 am and 04.00 pm to avoid diurnal anterior segment changes. The data was exported from the Pentacam and the CST. Additionally, the manifest refraction spherical equivalent (MRSE) was calculated by adding sphere and half of the cylinder power for each eye included (Table 2). Three groups (G1-G3) were formed according to age with G1 including children aged 3–10 years, G2 adolescents aged 11–20 years and G3 adults equal to or older than 21 years of age.

Tomographic outcome parameters included flat anterior (K1F) and posterior (K1B), steep anterior (K2F) and posterior (K2B) meridians, thinnest corneal thickness (TCT) and maximal anterior keratometry (Kmax). Biomechanically, DA ratio 2 mm, IR, ARTh, SP-A1, A1 velocity, CBI and BEST were assessed. Further parameters included the stress-strain-index (SSI) and the CST-derived IOP measurements. The SSI indicates the biomechanical strength of a healthy, 50-years-old patient’s cornea in a curve (value 1.0) and compares this value to the examination result of the examined cornea: A left shift of the curve and a value > 1.0 indicate a higher, and a right shift with values < 1.0 a lower corneal stiffness^18^. The CST IOP measurements include a noncontact-IOP measurement (IOPnct) and a biomechanically-corrected IOP calculation (bIOP).

The first analysis was an intergroup comparison of the outcome parameters between the three groups G1-G3. The Kruskal-Wallis one-way ANOVA and Dunn’s post test were used because the majority of the data was not normally distributed as revealed by the Shapiro-Wilk test. To counteract the effect of any potential bias caused by the arbitrary grouping based on the first two decades of life in G1 and G2, an additional continuous variable analysis with simple linear regression was performed secondly for the biomechanical parameters to analyze, whether age as an independent variable significantly influences corneal biomechanics as dependent variables. The third analysis was an age-dependent intragroup comparison of the outcome parameters between male and female volunteers based on an unpaired t test for normally distributed and the Mann–Whitney-U test for not normally distributed parameters. The calculations were performed using Prism5 software (GraphPad software Inc., San Diego, California, United States of America) and significant differences were assumed with *p* < 0.05.

## Results

The three groups consisted of 250 children (mean age eight years), 350 adolescents (mean age 15 years) and 100 adults (mean age 49 years, Table 1). The ratio between right and left eyes was balanced in all groups and the same applied for the gender ratio with a slight female preponderance in G2 (Table 1).

**Table 1:** Demographic characteristics of the participating volunteers.

	G1: children, 3–10 years	G2: adolescents, 11–20 years	G3: adults, > 21 years
*n* (eyes)	250	350	100
n (OS/OD)	122/128	179/171	50/50
n (male/female)	121/129	142/208	50/50
Age (mean ± SD)	7.7 ± 1.9	15.3 ± 2.9	48.7 ± 13.8
Age range (min; max)	3; 10	11; 20	23; 78

G1, children’s group from 3–10 years. G2, adolescents’ group from 11–20 years. G3, adults’ group above > 21 years. Mean ± SD, mean ± standard deviation. OS, oculus sinister/left eye. OD, oculus dexter/right eye.

**Fig. 1:**
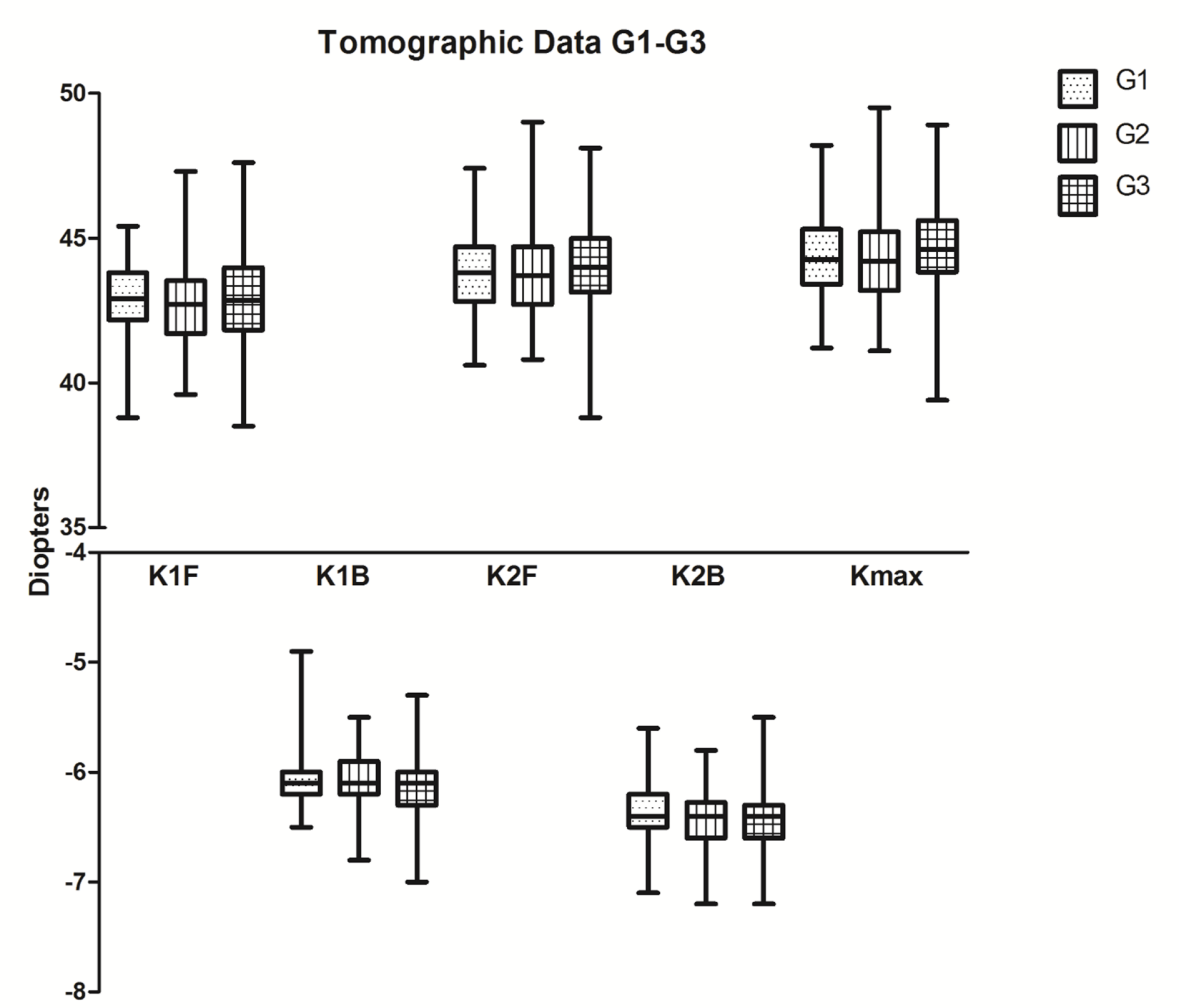
Tomographic data for Groups G1|G2|G3. Flat anterior (K1F) and posterior (K1B), steep anterior (K2F) and posterior (K2B) and maximal anterior keratometry (Kmax) values in diopters without statistically significant differences between G1 (250 children from 3–10 years), G2 (350 adolescents from 11–20 years) and G3 (100 adults > 21 years). Whiskers showing the range of the absolute values.

In view of the tomographic parameters K1F, K1B, K2F, K2B, TCT and Kmax, G1–G3 were comparable in the different age groups (*p* > 0.05, Table 2; Fig. 1) and the same applied for MRSE (*p* = 0.1906).

**Table 2:** Manifest refraction spherical equivalent and tomographic data of the participating volunteers.

	G1: children, 3–10 years	G2: adolescents, 11–20 years	G3: adults, > 21 years	*p*
MRSE (mean ± SD,[D])	0.3 ± 1.9	− 0.3 ± 1.6	0.0 ± 2.9	0.1906
MRSE range (min; max [D])	− 4.25; +4.4	− 4.25; +5.5	− 5.0; +5.5	
K1F (mean ± SD, [D])	42.9 ± 1.2	42.8 ± 1.4	42.9 ± 1.5	0.1281
K1F range (min; max [D])	38.8; 45.4	39.6; 47.3	38.5; 47.6	
K1B (mean ± SD, [D])	− 6.1 ± 0.2	− 6.1 ± 0.2	− 6.1 ± 0.2	0.2709
K1B range (min; max [D])	− 4.9; − 6.5	− 5.5; − 6.8	− 5.3; − 7.0	
K2F (mean ± SD, [D])	43.9 ± 1.3	43.8 ± 1.4	44.0 ± 1.4	0.922
K2F range (min; max [D])	40.6; 47.4	40.8; 49.0	38.8; 48.1	
K2B (mean ± SD, [D])	− 6.4 ± 0.2	− 6.4 ± 0.3	− 6.4 ± 0.3	0.1629
K2B range (min; max [D])	− 5.6; − 7.1	− 5.8; − 7.2	− 5.5; − 7.2	
Kmax [D]	44.4 ± 1.4	44.3 ± 1.4	44.6 ± 1.5	0.0514
Kmax range (min; max [D])	41.2; 48.2	41.1; 49.5	39.4; 48.9	
TCT [µm]	544 ± 32	547 ± 31	548 ± 31	0.2165
TCT range (min; max [µm])	476; 627	452; 626	469; 619	

G1, children’s group from 3–10 years. G2, adolescents’ group from 11–20 years. G3, adults’ group above > 21 years. Mean ± SD, mean ± standard deviation for manifest refraction spherical equivalent (sphere + 0.5 × cylinder power, MRSE), flat anterior (K1F) and posterior (K1B), steep anterior (K2F) and posterior (K2B) meridians, maximal anterior keratometry (Kmax) and thinnest corneal thickness (TCT). [D], diopters. P-values calculated by Kruskal–Wallis one-way ANOVA.

Biomechanically, DA ratio 2 mm and ARTh were comparable between G1-G3 (*p* > 0.7, Table 3).

**Table 3:** Biomechanical and intraocular pressure measurement data of the participating volunteers.

	G1: children, 3–10 years	G2: adolescents, 11–20 years	G3: adults, > 21 years	*p*	Dunn’s post test
DA ratio 2 mm (mean ± SD)	4.2 ± 0.4	4.2 ± 0.4	4.2 ± 0.4	0.918	
DA ratio 2 mm range (min; max)	3.3; 5.4	2.6; 5.5	2.9; 5.3		
ARTh (mean ± SD)	546.2 ± 112.6	537.8 ± 107.3	546.4 ± 137.1	0.7813	
ARTh range (min; max)	357.1; 898,1	308.3; 981.3	302.2; 1030.5		
SP-A1 (mean ± SD, [mmHg/mm])	108.4 ± 15.2	111.2 ± 16.2	114.8 ± 19.4	**0.0125**	G1 < G3
SP-A1 range (min; max, [mmHg/mm])	68.2; 151.5	74.2; 163.1	71.0; 163.6		
IR (mean ± SD, [1/mm])	7.3 ± 1.0	7.1 ± 1.2	7.4 ± 1.0	**0.0288**	G2 < G3
IR range (min; max, [1/mm])	4.6; 9.9	4.3; 10.8	5.4; 9.7		
A1 velocity (mean ± SD, [m/s])	0.132 ± 0.018	0.132 ± 0.018	0.139 ± 0.018	**0.0027**	G1 < G3 G2 < G3
A1 velocity range (min; max, [m/s])	0.092; 0.175	0.084; 0.178	0.103; 0.175		
SSI (mean ± SD)	1.2 ± 0.2	1.1 ± 0.2	1.3 ± 0.2	**< 0.0001**	G2 < G1< G3
SSI range (min; max)	0.6; 2.0	0.6; 1.7	0.8; 1.9		
CBI (mean ± SD)	0.3 ± 0.2	0.3 ± 0.2	0.2 ± 0.2	**0.001**	G1 > G3 G2 > G3
CBI range (min; max)	0.0; 0.9	0.0; 0.9	0.0; 0.9		
BEST (mean ± SD)	0.5 ± 0.5	0.5 ± 0.5	0.3 ± 0.5	**0.0015**	G1 > G3 G2 > G3
BEST range (min; max)	0.0; 2.0	0,0; 2.1	0.0; 2.3		
IOPnct (mean ± SD, [mmHg])	17.4 ± 2.9	17.5 ± 3.0	16.4 ± 2.7	**0.0006**	G1 > G3 G2 > G3
IOPnct range (min; max, [mmHg])	12.0; 25.5	12.0; 29.5	11.0; 26.5		
bIOP (mean ± SD, [mmHg])	17.3 ± 2.4	17.2 ± 2.4	15.2 ± 2.4	**< 0.0001**	G1 > G3 G2 > G3
bIOP range (min; max, [mmHg])	12.4; 24.7	11.8; 27.4	11.6; 22.9		

G1, children’s group from 3–10 years. G2, adolescents’ group from 11–20 years. G3, adults‘ group above > 21 years. Mean ± SD, mean ± standard deviation and range (minimum; maximum) for deformation amplitude ratio 2 mm (DA ratio 2 mm), Ambrósio relational thickness horizontal (ARTh), stiffness parameter A1 (SP-A1), integrated inverse radius (IR), A1 velocity, stress-strain-index (SSI), Corvis Biomechanical Index (CBI), Biomechanical E-Staging (BEST), non-contact intraocular pressure (IOPnct) and biomechanically corrected IOP (bIOP). P values calculated by Kruskal–Wallis one-way ANOVA with Dunn’s post test to identify significant differences (bold) between the individual groups G1-G3.

**Fig. 2:**
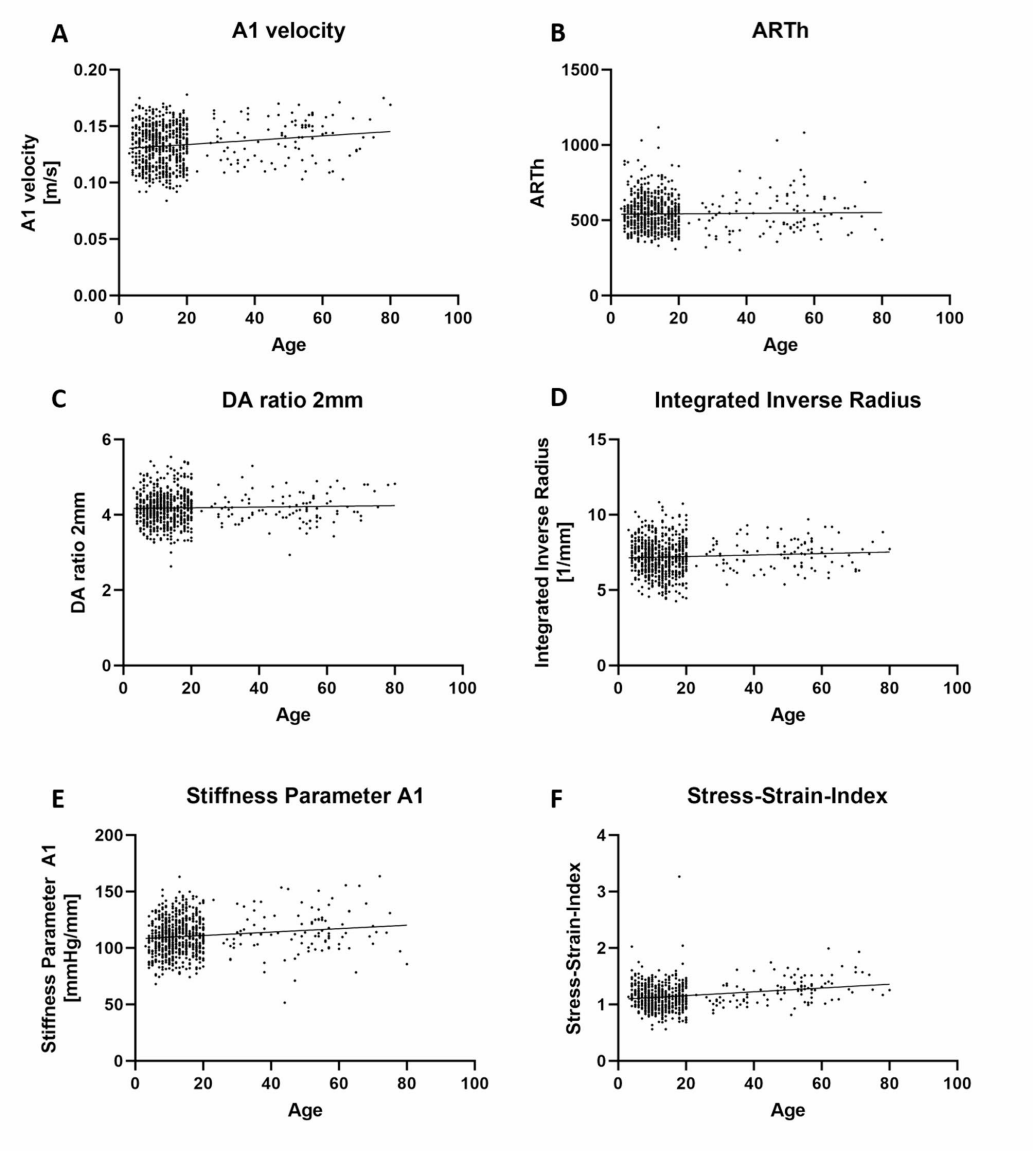
Continuous variable analysis with simple linear regression for biomechanical parameters according to age. **A** A1 velocity, velocity of the corneal apex at inward applanation. **B** ARTh, Ambrósio relational thickness horizontal, ratio of corneal thickness at thinnest corneal thickness to the pachymetric progression index. **C** DA ratio 2 mm, ratio between central deformation and deformation 2 mm from the corneal center. **D** IR, integrated inverse radius, sum of the inverse radii of the concave state between the first (A1) and second (A2) applanation. **E** Stiffness Parameter A1, ratio of the force of the air applied to the corneal displacement. **F** Stress–Strain-Index assessing biomechanical corneal stiffness. Regression slope significantly non-zero for A1 velocity (*p*<0.0001, **A**), SP-A1 (*p*=0.0004, **E**) and SSI (*p*<0.0001, **F**).

**Fig. 3:**
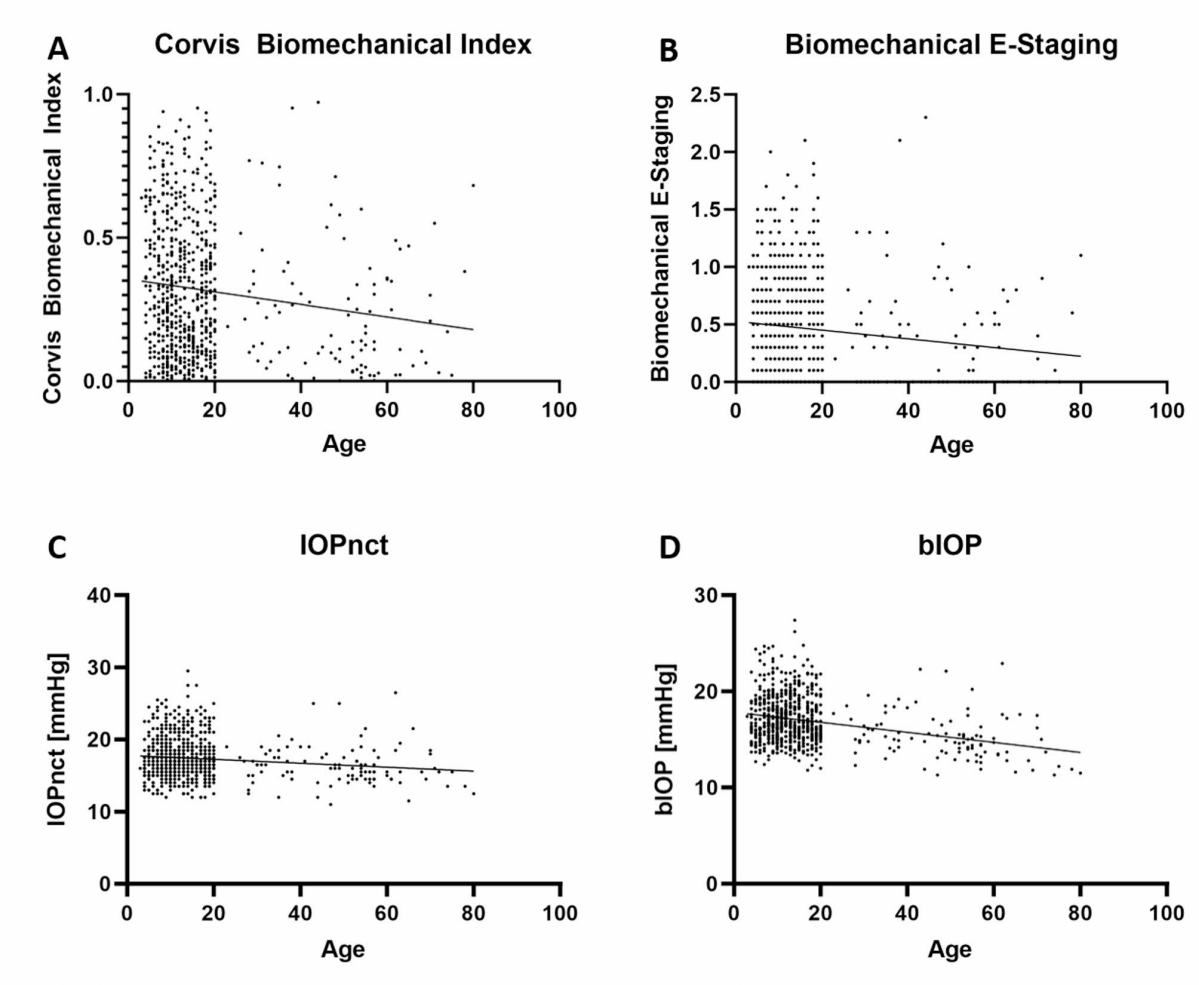
Continuous variable analysis with simple linear regression for combined biomechanical parameters and intraocular pressure values according to age. **A** Corvis Biomechanical Index (CBI). **B** Biomechanical E-Staging (BEST). **C** non-contact intraocular pressure (IOPnct). **D** biomechanically adjusted intraocular pressure (bIOP). Regression slope significantly non-zero for CBI (*p*=0.0004, **A**), BEST (*p*=0.0015, **B**), IOPnct (*p*=0.0003, **C**) and bIOP (*p*<0.0001, **D**).

Significant differences between G1–G3 were found for SP-A1, A1 velocity, SSI, CBI, BEST, IOPnct and bIOP (Table 3). Whilst SP-A1 increased statistically significantly with age, A1 velocity was significantly higher in adults than in both children and adolescents (Table 3). Adolescents showed a significantly lower SSI than both children and adults (Table 3). In contrast, adults showed a significantly lower CBI, BEST, IOPnct and bIOP than both children and adolescents (Table 3). In the continuous variable analysis, the simple linear regression slope was significantly non-zero for A1 velocity (*p* < 0.0001, Fig. 2A), SP-A1 (*p* = 0.0004, Fig. 2E),, SSI (*p* < 0.0001, Fig. 2F), CBI (*p* = 0.0004, Fig. 3A), BEST (*p* = 0.0015, Fig. 3B), IOPnct (*p* = 0.0003, Fig. 3C) and bIOP (*p* < 0.0001, Fig. 3D), whereas there was no significance for ARTh (*p* = 0.6352, Fig. 2B), DA ratio 2 mm (*p* = 0.3835, Fig. 2C) and IR (*p* = 0.0872, Fig. 2D).

The intragroup comparison between male and female volunteers aimed to analyze, whether gender influenced the biomechanical measurement results in the different age groups (Table 4).

**Table 4:** Gender-dependent biomechanical and intraocular pressure measurement data of the participating volunteers.

(a)	G1: children, 3–10 years		
	m = 121	f = 129	*p*
DA ratio 2 mm (mean ± SD)	4.2 ± 0.3	4.1 ± 0.4	0.2863^T^
DA ratio 2 mm range (min; max)	3.4; 5.1	3.3; 5.4	
ARTh (mean ± SD)	554.5 ± 113.7	538.3 ± 111.5	0.2192^M^
ARTh range (min; max)	357.1; 870.9	370.7; 898.1	
SP-A1 (mean ± SD, [mmHg/mm])	107.2 ± 14.9	109.4 ± 15.4	0.2533^T^
SP-A1 range (min; max, [mmHg/mm])	75.7; 146.2	68.2; 151.5	
IR (mean ± SD, [1/mm])	7.3 ± 1.0	7.2 ± 1.1	0.2685^T^
IR range (min; max, [1/mm])	5.2; 9.9	4.6; 9.6	
A1 velocity (mean ± SD, [m/s])	0.134 ± 0.018	0.131 ± 0.019	0.1863^M^
A1 velocity range (min; max, [m/s])	0.092; 0.168	0.092; 0.175	
SSI (mean ± SD)	1.1 ± 0.2	1.2 ± 0.2	0.4253^M^
SSI range (min; max)	0.6; 2.0	0.6; 1.8	
CBI (mean ± SD)	0.3 ± 0.2	0.3 ± 0.2	0.6841^M^
CBI range (min; max)	0.0; 0.9	0.0; 0.9	
BEST (mean ± SD)	0.5 ± 0.5	0.5 ± 0.5	0.4552^M^
BEST range (min; max)	0.0; 1.5	0.0; 2.0	
IOPnct (mean ± SD, [mmHg])	17.1 ± 2.7	17.8 ± 3.0	**0.0248** ^**M**^
IOPnct range (min; max, [mmHg])	12.5; 25.5	12.0; 25.5	
bIOP (mean ± SD, [mmHg])	17.0 ± 2.5	17.6 ± 2.4	**0.0351** ^**M**^
bIOP range (min; max, [mmHg])	12.4; 24.7	12.9; 24.4	

(b)	G2: adolescents, 11–20 years		
	m = 142	f = 208	*p*
DA ratio 2 mm (mean ± SD)	4.2 ± 0.4	4.2 ± 0.4	0.7518^M^
DA ratio 2 mm range (min; max)	3.0; 5.4	2.6; 5.5	
ARTh (mean ± SD)	555.2 ± 119.1	526.0 ± 97.1	**0.0337** ^**M**^
ARTh range (min; max)	352.7; 981.3	308.3; 865,3	
SP-A1 (mean ± SD, [mmHg/mm])	112.1 ± 16.2	110.5 ± 16.2	0.3620^T^
SP-A1 range (min; max, [mmHg/mm])	74.2; 149.9	77.5; 163.1	
IR (mean ± SD, [1/mm])	7.1 ± 1.2	7.1 ± 1.2	0.7828^T^
IR range (min; max, [1/mm])	4.3; 10.8	4.5; 10.7	
A1 velocity(mean ± SD, [m/s])	0.131 ± 0.019	0.133 ± 0.018	0.1765^M^
A1 velocity range (min; max, [m/s])	0.084; 0.167	0.092; 0.178	
SSI (mean ± SD)	1.1 ± 0.2	1.1 ± 0.2	0.2627^M^
SSI range (min; max)	0.7; 1.7	0.6; 1.7	
CBI (mean ± SD)	0.3 ± 0.2	0.3 ± 0.2	0.3239^M^
CBI range (min; max)	0.0; 0.9	0.0; 0.9	
BEST (mean ± SD)	0.4 ± 0.5	0.5 ± 0.5	0.2563^M^
BEST range (min; max)	0.0; 2.1	0.0; 1.8	
IOPnct (mean ± SD, [mmHg])	17.5 ± 3.0	17.6 ± 3.0	0.7727^M^
IOPnct range (min; max, [mmHg])	12.0; 29.5	12.0; 27.5	
bIOP (mean ± SD, [mmHg])	17.1 ± 2.5	17.2 ± 2.4	0.8828^M^
bIOP range (min; max, [mmHg])	12.0; 27.4	11.8; 26.2	

(c)	G3: adults, > 21 years		
	m = 50	f = 50	*p*
DA ratio 2 mm (mean ± SD)	4.2 ± 0.4	4.2 ± 0.4	0.4356^T^
DA ratio 2 mm range (min; max)	2.9; 4.9	3.4; 5.3	
ARTh (mean ± SD)	568.4 ± 144.4	524.4 ± 127.1	0.1234^M^
ARTh range (min; max)	370.4; 1030.5	302.2; 834.3	
SP-A1 (mean ± SD, [mmHg/mm])	114.6 ± 18.1	114.9 ± 20.7	0.9337^T^
SP-A1 range (min; max, [mmHg/mm])	71.0; 163.6	71.0; 155.6	
IR (mean ± SD, [1/mm]))	7.3 ± 0.9	7.5 ± 1.0	0.4092^T^
IR range (min; max, [1/mm])	5.4; 9.0	5.8; 9.7	
A1 velocity(mean ± SD, [m/s])	0.142 ± 0.019	0.137 ± 0.018	0.2038^T^
A1 velocity range (min; max, [m/s])	0.103; 0.175	0.103; 0.171	
SSI (mean ± SD)	1.2 ± 0.2	1.3 ± 0.3	0.3109^M^
SSI range (min; max)	0.8; 1.6	0.9; 1.9	
CBI (mean ± SD)	0.2 ± 0.2	0.3 ± 0.3	0.1961^M^
CBI range (min; max)	0.0; 0.7	0.0; 0.9	
BEST (mean ± SD)	0.3 ± 0.3	0.4 ± 0.5	0.3024^M^
BEST range (min; max)	0.0; 1.1	0.0; 2.3	
IOPnct (mean ± SD, [mmHg])	16.3 ± 2.7	16.4 ± 2.7	0.6731^M^
IOPnct range (min; max, [mmHg])	11.0; 25.0	11.5; 26.5	
bIOP (mean ± SD, [mmHg])	15.1 ± 2.5	15.3 ± 2.2	0.5281^M^
bIOP range (min; max, [mmHg])	11.3; 22.3	11.6; 22.9	

(a) G1, children’s group from 3–10 years, (b) G2, adolescents’ group from 11–20 years, (c) G3, adults‘ group above > 21 years with m=male and f=female volunteers. Mean ± SD, mean ± standard deviation and range (minimum; maximum) for deformation amplitude ratio 2 mm (DA ratio 2 mm), Ambrósio relational thickness horizontal (ARTh), stiffness parameter A1 (SP-A1), integrated inverse radius (IR), A1 velocity, stress-strain-index (SSI), Corvis Biomechanical Index (CBI), Biomechanical E-Staging (BEST), non-contact intraocular pressure (IOPnct) and biomechanically corrected IOP (bIOP). P-values calculated by unpaired t-test^T^ if normally distributed and by Mann-Whitney-U test if not normally distributed as assessed by Shapiro-Wilk test. Bold, significant differences.

With exception of the thickness-related parameter ARTh in G2, there was no significant biomechanical difference between males and females in G1, G2 and G3. The IOP measurements were significantly lower in males than in females in G1 (children, *p* < 0.04), but comparable between males and females in G2 and G3 (Table 4).

## Discussion

The current study investigated corneal biomechanical properties based on CST measurements in healthy eyes of Caucasians (250 children (3–10 years old), 350 adolescents (11–20 years old) and 100 adults (older than 21 years)) with comparable tomography data. The MRSE ranged between + 0.3 and − 0.3 diopters for all age groups without statistically significant difference (*p* = 0.1906). It can thus be considered as similar among the groups (Table 2), so that the study results were unlikely to have been influenced by differences in refraction. Tomographically, the three age groups were comparable based on an analysis of the parameters K1F, K1B, K2F, K2B, TCT and Kmax (Table 2; Fig. 1), which is important because corneal thickness has been reported to be a confounding factor in the assessment of corneal biomechanics. As the three age groups were tomographically comparable and composed of a balanced number of both (1) right and left eyes and (2) male and female volunteers, the key question was, whether, and if so, which dynamic corneal response CST parameters differed in dependence of age.

First, the measurement results for the parameters ARTh and DA ratio 2 mm were comparable between the three age groups G1-G3 (Table 3; Fig. 2B, C). The parameter ARTh is based on the corneal thickness profile in the temporal-nasal direction and, consequently, similar ARTh values in G1-G3 must have been expected because of the similar corneal tomography across all age groups. The DA ratio 2 mm is reported to be possibly confounded by scleral rigidity, retrobulbar fat and extraocular muscle tone which may influence the measurement results^19^.

The parameter SP-A1 increased significantly with age in the present study (Table 3; Fig. 2E) and was lowest in G1, higher in G2 and highest in G3, which is in line with the reported stiffening of the human cornea with age stated in the existing literature.

Two other studies investigated corneal biomechanics in healthy volunteers of different age. One study analyzed corneal biomechanical properties with the CST in five age groups of healthy Chinese individuals (11–20 (*n* = 105), 21–30 (*n* = 112), 31–40 (*n* = 113), 41–50 (*n* = 100), > 50 years old (*n* = 113)) and found (1) an upward trend of SP-A1 after the age of 30 years and (2) a statistically significant upward trend for SSI between the five age groups^11^. Also in the current study, the SSI was lowest in G2 and highest in G3 (Table 3), thus indicating a stiffer cornea in adults. In contrast to the current study, however, the aforementioned groups were not compared tomographically, there was no information stated about refractive errors of the participants and there was no age group including children younger or equal to ten years^11^.

The second study consisted of six age groups of healthy Indians (5–10 (*n* = 37), 11–20 (*n* = 113), 21–30 (*n* = 396), 31–40 (*n* = 116), 41–50 (*n* = 39), > 50 years old (*n* = 17)) and the groups were matched for central corneal thickness and IOP^17^. The authors reported that DA ratio 2 mm, bIOP and SP-A1 were significantly different, whilst ARTh, IR and CBI were similar in all age groups^17^. They excluded participants with a spherical equivalent refraction greater than or equal to six diopters, resulting in a mean spherical equivalent refraction of + 2.48 ± 2.06 diopters in 320 male participants and − 2.75 ± 2.12 diopters in 398 female participants^17^.

The current study found significantly higher IR values in G3 than in G2 (Table 3), but without a significant increase in the linear regression (Fig. 2D). Together with a comparable A1 velocity in G1 and G2 and a significantly higher value in G3 (Table 3), this would indicate a biomechanically weaker cornea in the adult group. Among healthy Indians, A1 velocity was comparable between the age groups and IR decreased with age^17^, thus indicating a corneal stiffening with age. In healthy Chinese individuals, there were comparable differences between the age groups to our study for A1 velocity (11–20 years: 0.136 ± 0.017 (current study: 0.132 ± 0.018) and 41–50 years: 0.139 ± 0.024 (current study: 0.139 ± 0.018)) and for IR (11–20 years: 8.689 ± 1.192 (current study: 7.1 ± 1.2) and 41–50 years: 8.743 ± 2.117 (current study: 7.4 ± 1.0))^11^. Consequently, these two parameters must be assumed to be the most susceptible to measurement fluctuations and therefore appear to be less suitable for detecting an increase in corneal stiffness in healthy individuals.

The CBI and the BEST are combined indices and both showed significantly higher values in G1 and G2 than G3 (Table 3; Fig. 3A and B), which indicates a higher corneal stiffness in adults. Similar to the results reported among healthy Indians^17^, this study found significantly higher values for the CST-derived IOP parameters in children and adolescents versus adults (Table 3; Fig. 3C, D), which is plausible as it has been reported that IOP decreases with age in healthy subjects^20^.

With the exception of ARTh in G2, all biomechanical parameters examined in the current study were comparable between male and female volunteers. Although there exist similar results reported from healthy Indians^17^ and this might indicate that gender plays a minor role in biomechanical corneal measurement differences of healthy subjects, other studies reported significant differences in corneal biomechanics according to age^8,13–15^. In contrast, the IOP measurements revealed significantly higher IOP values for IOPnct and bIOP in female as opposed to male children up to ten years (G1, Table 4). This finding is in line with other studies that reported significantly higher IOP values in 7-year-old and 12-year-old females when compared to males^21^. Another study reported sex to be the second most influential factor on the IOP in children, but concluded that the reason for higher IOP values in female compared to male children still remains unclear^22^. In both G2 and G3, IOP values were continuously higher in females than males, without reaching statistical significance (Table 4) which corresponds with other reports stating that mean IOP levels in females were consistently higher than in males from the pre-teens up to adult life^23^.

A comparative multicenter study compared corneal biomechanical properties in a healthy Chinese and Caucasian population matched according to age (Chinese: *n* = 503, 30 ± 6.8 years, Caucasians: *n* = 452, 31.1 ± 6.8 years, mean ± standard deviation), corneal thickness and bIOP and found significant differences for SP-A1, ARTh and SSI between the Chinese and Caucasian cohort^24^. Based on these ethnical variances in corneal biomechanical properties, differences could also exist between Chinese, Indian and Caucasian cohorts of other age. This is important to investigate, because a difference between ethnicities could play a role in disease diagnosis. Additionally, biomechanical screening parameters may help to differentiate between ectatic and healthy corneas, so that information about these parameters across different age groups may serve as a reference^17^. To the best of our knowledge, the current study is the first to investigate corneal biomechanical properties in a comparatively large group of Caucasian children and adolescents compared to adults.

The results indicate a significant increase in corneal stiffness (as measured by the parameters SP-A1, SSI, CBI and BEST) from children or adolescents to adults, whilst (1) ARTh and DA ratio 2 mm did not differ significantly and (2) A1 velocity and IR seem to be most prone to measurement fluctuations.

Whereas the comparatively large cohorts in G1 and G2, the comparable tomography and the mean MRSE between ± 0.3 diopters in all age groups represent strengths of the current study, it is not without limitations. First, the definition of the age groups was arbitrary with a focus on the first two decades of life in G1 and G2 potentially neglecting hormonal effects during puberty. Second, they were not matched according to Goldmann applanation-based IOP measurements, which may also be considered as a confounding factor for the measurement of corneal biomechanical properties^25^. Third, this study does not provide evidence to determine the mechanism of changing corneal biomechanical properties with ageing because of its cross-sectional character. This purpose has to be addressed with longitudinal healthy cohort follow-ups.

As there were statistically significant differences between healthy corneas according to age, it can be hypothesized that differences would also exist between comparable keratectasia severity stages in different age groups, which could influence biomechanical keratectasia severity staging and progression assessment.

## Conclusions

Identifying differences of corneal biomechanical properties in healthy subjects is of interest because of its increasing importance in the screening for ectatic corneal diseases and its implications on ophthalmologic treatment decisions (also in refractive surgery). The current study found small, yet statistically significant biomechanical differences in the corneal deformation response between healthy Caucasian children, adolescents and adults with comparable tomography values, thus adding a possible reference to the existing literature. In a next step, it has to be clarified, whether these age-related differences do also apply for keratectasia of comparable severity, thus possibly influencing CST measurement results and interpretation.

## Data Availability

Data available within the article or its supplementary materials.
